# ENSURING COMPETENCE: ADDRESSING THE NEED FOR LGBTQ+ AGING EDUCATION

**DOI:** 10.1093/geroni/igac059.866

**Published:** 2022-12-20

**Authors:** Aaron Guest

**Affiliations:** Arizona State University, Phoenix, Arizona, United States

## Abstract

The continued demographic shifts in population aging are coupled with an increase in self-identified, LGBTQ+ individuals entering older age. While they share some common age-related experiences with non-LGBTQ+ individuals, they also face unique challenges as they age, including additional intersectional pressures beyond their sexual or gender identity. These challenges are exacerbated by pre-existing health inequities and heteronormative social support systems. Of particular concern is the ability to locate affirming and accessible social and health services. Gerontologists have a critical role in assisting older LGBTQ+ individuals in navigating complex social systems. To do so requires they have the appropriate education to develop the cultural humility needed to understand and assist these individuals. It necessitates the engagement of gerontology and geriatric education programs in educating future gerontologist professionals about LGBTQ+ older individuals. Critically, it requires the identification of appropriate instructors who are equipped to educate on LGBTQ+ aging topics.

